# Extracellular Vesicles as a Potential Tool in Cancer Diagnosis and Therapy

**DOI:** 10.3390/biomedicines14071411

**Published:** 2026-06-23

**Authors:** Giovanni Citelli, Simone Peria, Sabina Di Matteo, Roberto Sirica, Federica Palmiero, Daniela Rita Vantaggiato, Rita Paola Debri, Raffaele Conte, Gianfranco Peluso

**Affiliations:** 1 Faculty of Medicine and Surgery, Saint Camillus International University of Health Sciences, Via di Sant’Alessandro 8, 00131 Rome, Italy; giovanni.citelli@unicamillus.org (G.C.); simone.peria@unicamillus.org (S.P.); sabina.dimatteo@unicamillus.org (S.D.M.); daniela.vantaggiato@unicamillus.org (D.R.V.); gianfranco.peluso@unicamillus.org (G.P.); 2 Department of Earth and Marine Science (DiSTeM), University of Palermo, 90133 Palermo, Italy; 3 IRCCS San Camillo Hospital, Via Alberoni, 70, 30126 Venice, Italy; 4 AMES Diagnostic Center, Via Padre Carmine Fico 24, 80013 Casalnuovo di Napoli, Italy; roberto.sirica@centroames.it; 5 Department of Biomedicine and Prevention, University of Rome “Tor Vergata”, Via Montpellier 1, 00133 Rome, Italy; federica.palmiero@uniroma2.it; 6 Research Institute on Terrestrial Ecosystems (IRET)-CNR, Via Pietro Castellino 111, 80131 Naples, Italy; 7 National Biodiversity Future Center (NBFC), 90133 Palermo, Italy

**Keywords:** extracellular vesicles, cancer diagnosis, liquid biopsy, drug delivery, plant-derived extracellular vesicles, extracellular vesicles isolation methods, cancer therapy

## Abstract

Cancer remains one of the leading causes of morbidity and mortality worldwide, with lung, breast, and colorectal cancers among the most prevalent and lethal malignancies. In recent years, extracellular vesicles (EVs) have emerged as important mediators of intercellular communication and promising tools in oncology. EVs are membrane-bound vesicles released by most cell types and carry diverse biomolecules, including nucleic acids, proteins, lipids, and metabolites derived from their parent cells. Their presence in biological fluids makes them attractive candidates for liquid biopsy applications and minimally invasive cancer diagnosis. In addition, EVs have gained considerable attention as therapeutic platforms due to their biocompatibility, stability, and ability to deliver functional cargo to recipient cells. Beyond mammalian EVs, plant-derived extracellular vesicles (PDEVs) are increasingly being investigated as scalable and potentially safe nanocarriers for biomedical applications. This review summarizes current advances in the use of EVs for cancer diagnosis and therapy, with particular emphasis on their role as biomarkers, drug-delivery systems, and emerging therapeutic agents. Furthermore, the review discusses current challenges and future perspectives related to EV isolation, characterization, and clinical translation in oncology.

## 1. Introduction

The human body contains approximately 30 billion cells that maintain physiological homeostasis through highly regulated molecular communication networks. When these regulatory mechanisms fail, uncontrolled cell proliferation may occur, leading to cancer [1]. Cancer originates from genetic alterations that may be inherited or induced by environmental factors. These changes lead to uncontrolled cell division and the formation of tumor masses. In advanced stages, malignant cells may migrate from the primary tumor and establish secondary tumors in distant tissues through a process known as metastasis [1]. Cancer represents a major global health challenge with profound social and economic consequences. According to recent estimates, around 20 million new cancer cases were reported worldwide in 2022, resulting in approximately 9.7 million deaths [2,3]. Epidemiological projections suggest that cancer incidence may reach 35 million new cases annually by 2050, reflecting population ageing and increased exposure to risk factors such as environmental pollution and lifestyle changes. The geographic distribution of cancer is irregular. Asia accounts for nearly half of global cases and more than half of cancer-related deaths, largely due to its large population. Europe follows with roughly 22% of cases, while North America accounts for about 13%. Incidence rates are generally higher in countries with a high Human Development Index [2,3]. Among all cancer types, lung cancer remains the leading cause of cancer mortality, followed by breast and colorectal cancers. Prostate and stomach cancers are also widely diagnosed worldwide. As cancer incidence increases, global expenditure on anticancer drugs continues to rise, while treatment efficacy is often limited by drug resistance, toxicity, and insufficient targeting [4]. Extracellular vesicles are membrane-bound particles released by both eukaryotic and prokaryotic cells. They were first identified in human plasma by Chargaff and West in 1946 [5], while in plants they were found in 1967 within the apoplastic space; however, their detailed characterization became possible only decades later with advances in electron microscopy [6,7]. According to the International Society for Extracellular Vesicles (ISEV), EVs can be broadly classified into “Small extracellular vesicles” (sEVs) with a diameter below 200 nm and “Large extracellular vesicles” (lEVs) with a diameter between 200 nm and 5 µm [8,9]. EVs derived from mammalian cells have been extensively investigated because of their direct involvement in human physiology and disease. Tumor cells also release EVs, which participate in complex communication within the tumor microenvironment [10]. In addition to mammalian EVs, plant-derived extracellular vesicles are receiving increasing attention. These vesicles are attractive because they can be obtained in large quantities and are generally considered safe due to the long history of human exposure to plant-derived compounds. PDEVs include several subclasses: “Exosome-like vesicles” (30–150 nm) originating from multivesicular bodies, “Microvesicles” (100–1000 nm) formed by plasma membrane budding and “Apoptotic bodies” (1000–5000 nm) generated during programmed cell death [10,11,12]. Another plant-specific vesicle type derives from “exocyst-positive organelles” (EXPOs), structures approximately 200–500 nm in size that may contain ribosomes [13,14].

EVs act as carriers of biologically active molecules. Their cargo includes DNA and RNA (miRNA, siRNA, piRNA), proteins, lipids and secondary metabolites. Through these molecules, EVs participate in cell-to-cell communication, immune modulation, defense responses, and maintenance of tissue homeostasis [15]. Because of their composition and biological properties, EVs exhibit a range of activities including antioxidant, anti-inflammatory, antimicrobial, and antitumor effects. Their natural biocompatibility, low immunogenicity, and ability to cross biological barriers, including the blood–brain barrier, make them particularly interesting for biomedical applications [16].

This review summarizes the current knowledge on the use of extracellular vesicles in cancer treatment, highlighting their potential as both diagnostic and therapeutic agents. It also discusses emerging sources for EV procurement and explores possible new functions that could further expand their role in oncology [17,18,19,20,21]. Unlike most existing reviews, which predominantly focus on mammalian EVs, this work provides an integrated comparison between mammalian-derived vesicles and plant-derived extracellular vesicles, highlighting the latter as an emerging and underexplored resource with advantages in safety, scalability, and intrinsic bioactivity. In addition, this review goes beyond descriptive biology by focusing on the translational potential of EVs as drug delivery systems, addressing key challenges such as targeting, loading efficiency, and clinical applicability. A further element of novelty lies in the emphasis on EVs as active nanoplatforms, whose cargo—particularly plant secondary metabolites and regulatory RNAs—may exert direct therapeutic effects rather than acting solely as carriers. By combining comparative analysis, emerging vesicle sources, and a strong translational perspective, this review offers a more comprehensive and forward-looking contribution than currently available literature.

## 2. Extracellular Vesicle Isolation Methods

Extracellular vesicles can be isolated from both mammalian and plant sources through a variety of methodologies that differ substantially in terms of yield, purity, scalability, preservation of vesicle integrity, and suitability for downstream characterization and translational applications. Current isolation approaches are described in the MISEV2023 guidelines established by the International Society for Extracellular Vesicles, which emphasize the importance of selecting isolation procedures according to the intended biological and clinical application rather than relying on a single universally optimal method [8,22]. EVs have been successfully recovered from a wide range of biological sources, including animals, bacteria, fungi, and plants. In plant systems, vesicles have been isolated from apoplastic fluids, fruits, leaves, roots, pollen, seeds, flowers, bulbs, whole plant extracts, cell cultures, root cultures, and hydroponic exudates, highlighting the versatility of PDEVs as emerging platforms for biomedical applications [23,24,25]. Among the available methodologies, differential ultracentrifugation (dUC) remains the most widely used and historically established approach for EV isolation [8,26]. In plant-derived samples, vesicles are typically obtained from apoplastic fluid or tissue homogenates and subsequently subjected to sequential centrifugation steps at increasing centrifugal forces to remove cellular debris and progressively enrich EV populations [27]. The widespread adoption of dUC is primarily attributable to its relatively low cost, accessibility, and ability to generate reproducible EV preparations. Nevertheless, the method presents several limitations that may affect both EV characterization and translational development. Co-isolation of proteins and other particles with similar sedimentation properties can compromise purity, while prolonged exposure to high centrifugal forces may induce vesicle aggregation, membrane deformation, or loss of bioactive cargo [28]. Consequently, dUC is increasingly regarded as a primary enrichment strategy rather than a definitive purification method and is frequently combined with secondary purification approaches such as size-exclusion chromatography (SEC) or density gradient centrifugation (DGC) to improve sample quality [29]. SEC has emerged as one of the most attractive complementary methods because it separates particles according to hydrodynamic size while minimizing mechanical stress on vesicles [28]. Larger vesicles elute rapidly, whereas smaller contaminants are retained within the porous matrix. Compared with dUC, SEC generally provides EV preparations with lower protein contamination and better preservation of vesicle morphology and functionality, features that are particularly important when accurate molecular characterization or therapeutic applications are required. However, SEC often suffers from limited throughput and scalability, restricting its application in large-scale production settings despite its suitability for analytical and preclinical studies [28]. Similarly, DGC represents a valuable strategy for obtaining highly purified and homogeneous EV populations [29,30]. By exploiting differences in buoyant density through sucrose-, iodixanol-, or iohexol-based gradients, DGC enables the separation of vesicles from contaminating proteins, organelles, and other nanoparticles. Gradients between 30% and 45% have been reported as particularly effective for PDEV isolation [29,30]. Because of its high resolving power, DGC is frequently employed when detailed characterization of EV subpopulations is required, including proteomic, lipidomic, and transcriptomic analyses. However, the increased purity achieved by DGC is often accompanied by lower recovery rates, longer processing times, and potential vesicle damage associated with repeated ultracentrifugation steps, thereby limiting its applicability for large-scale translational manufacturing [30]. Polymer-based precipitation methods, particularly those employing polyethylene glycol (PEG), have gained considerable attention as rapid and scalable alternatives [31]. These approaches typically achieve higher recovery yields than SEC or DGC and require minimal specialized equipment [32,33]. However, precipitation methods often co-isolate non-vesicular proteins, lipoproteins, and other macromolecular contaminants, reducing preparation purity and potentially interfering with downstream molecular analyses [34]. Consequently, although PEG-based isolation may be advantageous for large-scale exploratory studies, additional purification steps are frequently required before clinical or pharmaceutical applications [35]. The increasing interest in EV-based therapeutics has stimulated the development of advanced separation technologies aimed at improving both EV characterization and manufacturing scalability [36]. Field-flow fractionation (FFF) and free-flow electrophoresis (FFE) represent important examples of this technological evolution. Unlike conventional chromatographic methods, these techniques do not rely on a stationary phase, thereby reducing vesicle damage and facilitating the recovery of heterogeneous EV populations [37]. In asymmetric flow FFF, vesicles are separated according to their diffusion behavior and hydrodynamic size, allowing the generation of relatively homogeneous fractions suitable for detailed physicochemical characterization. Nevertheless, FFF alone cannot distinguish between biologically distinct particle populations and is therefore commonly combined with complementary purification approaches. In contrast, FFE separates particles according to surface charge and isoelectric properties, providing higher resolution and enabling the isolation of specific EV subpopulations [37]. This capability is particularly relevant for translational applications because it may facilitate the identification of vesicle subsets associated with specific biological functions, disease states, or therapeutic activities [38]. Affinity-based and ion-exchange approaches further extend the possibilities for selective EV isolation. By exploiting antibodies, aptamers, peptides, lectins, or other ligands directed against membrane-associated markers, these techniques enable enrichment of vesicle populations characterized by defined molecular signatures [39]. Such selectivity is highly valuable for precision diagnostics, biomarker discovery, and targeted drug delivery applications. However, these approaches remain limited by high costs, potential loss of EV heterogeneity, and the possibility of co-isolating non-vesicular particles expressing similar markers [39]. Alternative isolation strategies have also been proposed to address challenges associated with plant tissues. Enzymatic digestion, for example, has been successfully employed to facilitate vesicle release from sources characterized by rigid cell walls [40]. The controlled degradation of cellulose, hemicellulose, and pectin using specific enzyme mixtures enhances EV recovery while reducing contamination by intracellular components. Importantly, the milder extraction conditions may preserve vesicle integrity and bioactive cargo more effectively than aggressive mechanical disruption procedures, potentially improving the quality of EV preparations intended for therapeutic applications [40]. Hydrophobic interaction chromatography (HIC) has recently emerged as a simple and low-cost approach for EV isolation from plant-derived matrices [41]. By exploiting hydrophobic interactions between vesicle membranes and chromatographic substrates, HIC enables rapid EV enrichment with minimal instrumentation requirements. These characteristics make the technique particularly attractive for laboratories with limited resources and potentially for industrial implementation. Nevertheless, concerns remain regarding contaminant co-isolation, reproducibility, and the difficulty of distinguishing true extracellular vesicles from intracellular vesicles released during tissue processing [41]. Among emerging technologies, tangential flow filtration (TFF) is attracting particular attention because of its strong translational potential [42]. Unlike conventional dead-end filtration systems, TFF minimizes membrane fouling by directing sample flow parallel to the filtration membrane, thereby improving process efficiency and preserving vesicle integrity. The method is readily scalable, compatible with good manufacturing practice (GMP) requirements, and capable of processing large sample volumes, making it one of the most promising technologies for future industrial production of EV-based therapeutics [42]. Overall, the comparative evaluation of existing isolation methodologies demonstrates that no single technique simultaneously maximizes yield, purity, scalability, reproducibility, and preservation of vesicle functionality. Consequently, the choice of isolation strategy should be guided by the intended downstream application. High-purity approaches such as SEC and DGC are generally preferred for detailed EV characterization and biomarker discovery studies, whereas scalable technologies such as TFF and selected precipitation-based methods may be more suitable for large-scale manufacturing. Emerging affinity-based and electrophoretic approaches further support the development of precision EV therapeutics through the selective enrichment of biologically relevant vesicle subpopulations. Despite substantial technological progress, the lack of standardized isolation and characterization workflows remains a major obstacle to cross-study comparability and clinical translation. Future efforts should therefore focus on harmonizing methodological practices while integrating robust physicochemical and functional characterization criteria to accelerate the development of EV-based diagnostic and therapeutic platforms [8,26]. Table 1 recaps the described methods for EV isolation.

## 3. Extracellular Vesicles as Biomarkers for Cancer

**Figure 1:**
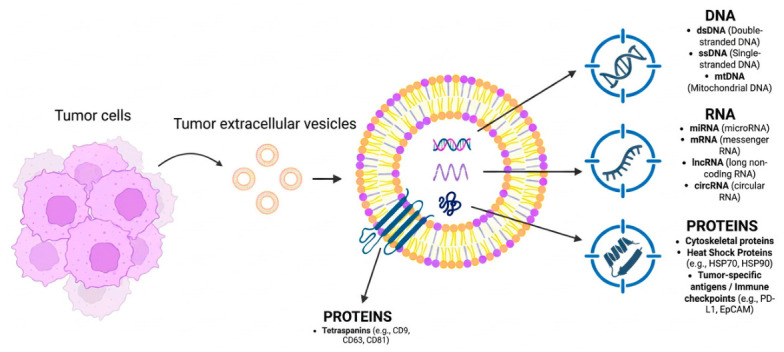
The image illustrates the release of extracellular vesicles by tumor cells into the extracellular environment. These EVs carry proteins on the membrane (tetraspanins) and a cargo that reflects their cell of origin. As depicted, the cargo of EVs includes DNA (such as double-stranded DNA, single-stranded DNA, and mitochondrial DNA), various types of RNA (including long non-coding RNA, circular RNA, and microRNA), proteins (such as cytoskeletal proteins, heat shock proteins, and antigens), as well as metabolites.

Early detection is critical for successful cancer treatment, and although tissue biopsy is currently the most widely used diagnostic method, its reliability can be limited because it samples only a small portion of the tumor [42]. For this reason, increasing attention has been directed toward liquid biopsy as a less invasive alternative. In this context, extracellular vesicles are emerging as promising biomarkers for cancer detection (Figure 1), since they carry a wide range of tumor-related molecules, including RNAs, proteins, DNA, and lipids that reflect the molecular profile of their cells of origin [42,43].

Evidence supporting the diagnostic value of EVs has been reported in several cancers.

### 3.1. Clear-Cell Renal Cell Carcinoma

In patients with clear-cell renal cell carcinoma (ccRCC), tumor-derived EVs positive for the epithelial cell adhesion molecule (EpCAM) were found in serum and showed significantly higher levels of miR-210 and miR-1233 compared with vesicles from healthy individuals. The study also demonstrated differences in expression between patients and controls, with miR-210 showing a sensitivity and specificity of 70% and 62%, respectively, and miR-1233 showing values of 81% and 76%. These findings suggest that these miRNAs could serve in the diagnosis of ccRCC through liquid biopsy [43,44,45]. Additional support for this approach was provided by Wu et al. [46], who analyzed RNA profiles in EVs from tumor tissue (Ti-EVs) and urine (uEVs). Their study included Ti-EVs from 25 patients and uEVs from 178 individuals, with urine samples collected before and after tumor removal. Prior to surgery, several RNAs—NDUFA4L2, SERPINA1, VEGFA, EGLN3, CPE, and C6orf223—were strongly expressed both in Ti-EVs from low-grade ccRCC and in uEVs from affected patients, forming a diagnostic panel with an area under the curve (AUC) of 0.922. For high-grade ccRCC, the RNAs NDUFA4L2, APOC1, TGFBI, and LINC00887 were similarly enriched, generating a diagnostic panel with an AUC of 0.874. After tumor removal, the levels of these markers in uEVs decreased markedly, further supporting the use of urinary EVs for early diagnosis and disease monitoring [46].

### 3.2. Breast Cancer

Breast cancer (BC) diagnosis still relies mainly on mammography, which can produce false negatives and may fail to detect small lesions, especially in early stages [47,48,49]. Several circulating biomarkers have also been investigated, but their sensitivity for early detection remains limited [50]. In this scenario, sEVs represent a promising alternative. Lee et al. identified the protein EDIL3 in circulating sEVs released by tumor cells and validated it as a biomarker associated with metastasis [51]. Other proteins present on the vesicle surface may also help distinguish tumor subtypes. For example, fibronectin—an adhesion-related molecule—has been proposed as a marker capable of differentiating BC derived from estrogen receptor-positive (ER+) or estrogen receptor-negative (ER−) cells [52,53]. Similarly, the transmembrane protein CD47, which functions as an inhibitory signal to macrophages and prevents immune-mediated clearance, has been detected on tumor-derived exosomes. Kibria et al. observed that BC patients had a significantly lower proportion of CD47-positive sEVs (0.7%) compared with healthy individuals (10%), suggesting a potential diagnostic role for this marker [54]. Beyond proteins, several miRNAs carried by tumor-derived sEVs—either overexpressed or downregulated—have also been proposed as diagnostic markers for BC [55].

### 3.3. Pancreatic Cancer

Pancreatic cancer has also been widely investigated in this context. One study compared DNA contained in sEVs with circulating cell-free DNA released by necrotic tumor cells, focusing on mutations in the KRAS gene, which regulates cell proliferation. Analysis of sEV-derived DNA allowed the detection of KRAS mutations in 66.7% of patients with localized disease and 85% of those with metastatic disease. In contrast, circulating cell-free DNA identified mutations in only 45.5% of localized cases and 57.9% of metastatic cases [56]. Additional biomarkers have also been described, including the proteins G protein-coupled receptor class C group 5 member C (GPRC5C) and epidermal growth factor receptor pathway substrate 8 (EPS8), both of which are enriched in sEVs from pancreatic cancer patients [57]. RNA molecules may also contribute to diagnosis. For instance, Takahashi et al. [58] reported that the long noncoding RNA HULC (Highly Upregulated in Liver Cancer) is highly expressed in tumor-derived sEVs. HULC promotes tumor cell migration and invasion by inducing epithelial–mesenchymal transition (EMT), a process that enables epithelial cells to acquire invasive mesenchymal characteristics [58].

### 3.4. Lung Cancer

Lung cancer represents another area where sEV-based biomarkers have shown promise. This disease is broadly divided into non-small cell lung cancer (NSCLC), the most common form, and the more aggressive small cell lung cancer (SCLC). Wan et al. [59] compared the diagnostic value of circulating cell-free DNA with that of DNA carried by sEVs released from NSCLC cells, focusing on mutations in the epidermal growth factor receptor (EGFR) gene. Their results showed that, particularly in early stages, sEV DNA displayed higher sensitivity and specificity for tumor detection than circulating DNA. Sensitivity values were 25.7% for sEV DNA compared with 14.2% for cell-free DNA, while specificity reached 96.6% and 91.7%, respectively. These findings suggest that analyzing sEV DNA may improve early detection, although the advantage becomes less pronounced in advanced disease stages [59]. In another study, Wang et al. [60] analyzed sEVs isolated from pleural fluid to distinguish between lung adenocarcinoma (APE), pleural tuberculosis (TPE), and benign pleural lesions (NPE). Several miRNAs were significantly overexpressed in APE, including miR-205-5p, miR-483-5p, miR-375, members of the miR-200 family, and miR-203a-3p. In TPE, miR-148a-3p, miR-451a, and miR-150-5p were enriched, whereas the same miRNAs were also detected in NPE but at different expression levels. Notably, miR-200 family members and miR-205 are already known to contribute to tumor progression and metastasis, highlighting their diagnostic potential [60]. Further work by Cazzoli et al. [61] evaluated miRNAs contained in tumor-derived sEVs and proposed different panels for screening and diagnosis of lung cancer. For screening, miR-378a, miR-379, miR-139-5p, and miR-200b-5p showed a sensitivity of 97.5% and specificity of 72%. For diagnosis, miR-151a-5p, miR-30a-3p, miR-200b-5p, miR-629, miR-100, and miR-154-3p achieved a sensitivity of 96% and specificity of 60%. These results indicate that sEV-associated miRNAs may help reduce both false positives and false negatives in lung cancer detection [61]. Other studies have examined the relationship between sEV cargo and tumor recurrence. Munagala et al. [62] analyzed tumor (H1299) and normal (Beas-2b) lung cells and found that the levels of miR-21 and miR-155 were significantly higher in tumor cells and in the sEVs they released. In models of recurrent lung cancer in nude mice, these miRNAs remained elevated in recurrent and relapsed tumors. In addition, vesicles circulating in serum contained increased levels of vascular endothelial growth factor (VEGF) and matrix metalloproteinase-2 (MMP-2), proteins associated with metastasis. These observations suggest that both miRNAs and proteins within sEVs may serve as markers for tumor recurrence and metastatic potential [62]. The use of multiple protein markers simultaneously may further improve diagnostic accuracy. In a study analyzing 30 proteins present in circulating sEVs—including CD81, TAG72, TSG101, CD9, EGFR, MUC1, NY-ESO-1, and CD151—in 109 patients with NSCLC and 110 healthy controls, the combined analysis achieved an overall diagnostic accuracy of 75.3% [63]. Additional work has shown that sEVs derived from saliva and lung tumor cells share several proteins, including BPIFA1, CRNN, MUC5B, and IQGAP, suggesting that both sources could be used for non-invasive diagnostic tests [64].

### 3.5. Head and Neck Cancers

EVs also play a role in diagnosing tumors affecting children. Pediatric high-grade glioma (pHGG) accounts for approximately 20% of childhood brain tumors. In a study analyzing sEVs released by several pHGG-derived cell lines (SJ-HGGx6, SJ-HGGx42, IFF-BT-150, and IFF-BT-173), researchers focused on vesicles secreted by RG-like glioma cells, since these tumors often contain cells resembling radial glia. The analysis revealed that sEVs released from RG-like glioma cells were positive for the dual marker GLAST+/PTPRZ1+, whereas vesicles released by healthy astrocytes showed almost no expression of this marker combination [65]. Oza et al. [66] found that pediatric diffuse midline gliomas (pDMG) with the H3K27M mutation exhibit strong intratumoral heterogeneity and intrinsic resistance to radiotherapy, partly driven by sEVs. Their study showed that sEVs released by radioresistant tumor cells are taken up by other glioma cells and can transfer a radioprotective phenotype. These sEVs carry proteins, miRNAs, and metabolites linked to glycolysis, oxidative phosphorylation, and DNA repair pathways. Upon uptake, they reprogram recipient cells by altering gene expression and metabolic activity, enhancing DNA repair capacity and improving survival after radiation exposure. Overall, the findings highlight sEV-mediated intercellular communication as a key mechanism of radiation resistance in H3K27M pDMG and a potential therapeutic and diagnostic target [66]. These findings suggest a potential application of sEVs as diagnostic markers detectable through liquid biopsy. With further validation, this approach might complement or partially replace magnetic resonance imaging, which is currently used to diagnose these tumors but can be complex to apply in pediatric patients. Similarly, in glioblastoma—a highly aggressive and infiltrative brain tumor characterized by poor prognosis and limited therapeutic options—the analysis of circulating sEVs has emerged as a promising non-invasive diagnostic approach. Due to the intrinsic heterogeneity of glioblastoma and the difficulty in obtaining representative tissue samples, conventional biopsy often fails to capture the full molecular landscape of the tumor. In this context, liquid biopsy strategies based on EVs offer a valuable alternative, as these vesicles can cross the BBB and carry tumor-specific molecular signatures into the systemic circulation. In a study involving tumor tissues and serum samples from 25 patients, along with serum from 30 healthy controls, the detection of mutant EGFR mRNA (EGFRvIII)—a well-known oncogenic variant frequently associated with glioblastoma—was evaluated. The results demonstrated that serum-derived sEVs could serve as a source of clinically relevant genetic information. Although serum-based analysis identified a lower proportion of patients (28% of 25) compared to tissue-based detection (47% of 30), it notably succeeded in detecting two cases that were false negatives by conventional biopsy [67]. This finding highlights the complementary role of EV-based diagnostics in overcoming sampling bias and tumor heterogeneity. Moreover, the presence of EGFRvIII mRNA within circulating sEVs underscores the stability of vesicle-associated nucleic acids, which are protected from enzymatic degradation in the bloodstream. This enhances their reliability as biomarkers for disease detection and monitoring. Beyond diagnosis, such approaches may also enable longitudinal assessment of tumor evolution, treatment response, and early detection of recurrence [67]. Similarly, Aibaidula et al. [68] isolated EVs carrying a wide range of tumor-associated molecules, including miRNAs (e.g., miR-21, miR-15b-3p), mRNAs such as EGFRvIII, circRNAs, and proteins like CD44 and GFAP. EV-based molecular signatures have shown high diagnostic performance, with reported sensitivities of 87–100% and specificities of 73–100%. Moreover, they described advanced methods to isolate tumor-derived EV subpopulations such as SEC-CD44 immunoprecipitation, microfluidics, and 5-ALA/PpIX-based enrichment [68]. Del Bene et al. [69] found that extracellular vesicles overcome key limitations of traditional liquid biopsy approaches in glioblastoma, including those related to circulating tumor cells (CTCs) and cell-free nucleic acids (cfNAs). Unlike CTCs, which are extremely rare, unstable, and poorly standardized across studies, EVs are consistently detectable and better reflect whole-tumor biology. Similarly, cfNAs—particularly cfDNA, which is scarcely detectable in glioblastoma—suffer from low sensitivity and high analytical and preanalytical complexity. These limitations are further compounded by the blood–brain barrier and the extensive intratumoral heterogeneity characteristic of glioblastoma. In contrast, EVs carry protected molecular cargo, including nucleic acids and proteins derived from tumor cells. Importantly, they can cross the blood–brain barrier and are enriched in glioblastoma patients, showing disease specificity. Their stability and integrative molecular content allow a more accurate representation of tumor status than single-analyte approaches [69].

### 3.6. Cancer of the Reproductive System

Prostate cancer (PCa) is the most common cancer in men and the second leading cause of cancer-related death after lung cancer [70]. The diagnostic potential of EV-derived markers has already led to the development of the first FDA-approved test based on EVs, the ExoDx Prostate IntelliScore (EPI). This assay detects three RNA biomarkers—ERG, Prostate Cancer Antigen 3 (PCA3), and SAM pointed domain containing Ets transcription factor (SPDEF)—contained in EVs released by tumor cells, enabling early and non-invasive detection of prostate cancer [71]. Moreover, a study conducted by Ferre-Giraldo et al. [72] found that semen-derived sEV content, in particular transcripts, can be used to predict aggressiveness of prostate cancer. Moreover, Liao et al. [73] reported that EVs play a key role in castration-resistant prostate cancer (CRPC), both as mediators of disease progression and as promising diagnostic biomarkers. They showed that PCa cells can develop resistance to androgen deprivation therapy (ADT) through EV-mediated intercellular communication, in which microRNAs, long non-coding RNAs, and proteins are transferred to neighboring cells, promoting the castration-resistant phenotype. EVs also facilitate crosstalk between tumor cells and the surrounding immune and stromal microenvironment, thereby accelerating CRPC progression. In addition, the release of diverse EV cargo molecules is considered a relevant mechanism underlying ADT resistance. The authors further emphasized the diagnostic potential of EVs in CRPC, highlighting their advantages over traditional liquid biopsy approaches based on circulating tumor cells and circulating tumor DNA. Although CTCs and ctDNA are widely studied as cancer biomarkers, their clinical application is limited by low abundance, short stability, and insufficient detection sensitivity. In this context, EVs represent a more stable and information-rich source of tumor-derived material [73]. Ovarian cancer is one of the major causes of death among women living in transitioning countries [74]. EVs may play a role in early diagnosis. Wang et al. [75] have found that EVs released by ovarian cancer cells and isolated from plasma and malignant ascites contain distinct miRNAs (including miR-1246, miR-1290, miR-483, miR-429, miR-34b-3p, miR-34c-5p, miR-145-5p, and miR-449a) [75]. These miRNAs contribute to cancer cell migration and invasion and have the potential to serve as biomarkers for early diagnosis [75]. Preußer et al. [76] provided the first proteomic profile of EVs derived directly from primary ovarian cancer tumor spheroids. Using a 3D spheroid model established from patient ascites, they isolated EVs through differential ultracentrifugation and size-exclusion chromatography and characterized them by nanoparticle tracking analysis, nanoflow cytometry, and electron microscopy before high-resolution mass spectrometry. Their analysis identified cancer-associated EV proteins, including danger-associated molecules linked to poor prognosis, as well as novel candidates such as RAB14, SCAMP3, and FAM3C, whose abundance correlated with progression-free survival. Integration with public databases further highlighted a tumor-specific protein signature (CORO1B, LAMP2, MSLN) differentially expressed in ovarian cancer compared to healthy controls [76]. Another recent study demonstrated that EVs isolated from ascites fluid in ovarian cancer patients exhibited high expression of a bacterial-derived protein (P15636_Protease1). This protein could potentially be used for the early diagnosis of the disease [77].

### 3.7. Good Manufacturing Practice and Clinical Trials

Considering the stringent regulations required for FDA approval of a drug—such as compliance with Good Manufacturing Practice (GMP)—it is unsurprising that so few EV-based markers and treatments are currently available. These regulations are designed to ensure the safety, quality, and purity of pharmaceuticals [78]. Among the main problems, the most critical for the reproducibility of biomarker recognition is the lack of a standardized isolation method to obtain EVs from complex biofluids such as plasma and tumor microenvironment [79]. In a recent study conducted by Humbert et al., the authors focused on the feasibility of obtaining a secretome rich in GMP-compliant EVs from cardiac progenitor cells [80]. Their findings demonstrate that it is indeed possible to isolate such EVs from complex biological samples while maintaining high standards of quality, repeatability, and compatibility, suggesting that similar scalability could help address the need for biomarker standardization [80].

Currently, several clinical trials are investigating the use of EVs as biomarkers. Typing the word cancer in the “Condition/disease” box, Biomarker in the “Other terms” box, and Extracellular vesicles in the “Intervention/treatment” box on the ClinicalTrials.gov website yielded 28 studies that attempted to understand whether EVs can be used as a diagnostic marker. These studies are summarized in Table 2.

However, several limitations must be considered when using EVs as diagnostic markers in liquid biopsy. First, there is a lack of standardized methods for EV isolation and characterization. Isolating EVs in large quantities increases heterogeneity, complicates analyses, and reduces specificity, which compromises the potential to establish a cause–effect relationship. In some cases, the material required to identify EVs may be insufficient, which can impact analysis times; additionally, the EV content may not accurately reflect the solid tumor profile. Variations in sample storage and transport methods, as well as possible contamination with proteins and lipoproteins [81], can lead to sample artifacts and introduce further variability. Ultimately, most clinical studies have been conducted on small patient cohorts, which limits the statistical power and reliability of the results [82]. Furthermore, before an EV biomarker is considered a biomarker, it must pass a series of validations. These include analytical and clinical validation. Analytical validation evaluates an assay’s performance characteristics, including accuracy, precision, analytical sensitivity and specificity, linearity, and range. Clinical validation, on the other hand, evaluates diagnostic accuracy and predictability through the analysis of thousands of samples [83].

Nonetheless, the International Society for Extracellular Vesicles guidelines (MISEV) are contributing to standardizing the field and improving the reliability of EV-based studies. Because EVs can be readily isolated from accessible biological fluids and accurately reflect the molecular profile of tumor cells, they represent a promising, minimally invasive alternative to conventional diagnostic approaches such as tissue biopsy and imaging techniques. Consequently, their application may significantly enhance early cancer detection, enable more precise patient stratification, and improve the monitoring of disease progression and recurrence (Table 3).

## 4. Extracellular Vesicles as Carriers for Cancer Drug Delivery

In recent years, EVs have attracted considerable interest as potential carriers for drug delivery in cancer therapy and other diseases. Their biological characteristics make them particularly suitable for this purpose. EVs show low immunogenicity, high specificity, and natural stability in biological fluids. They are also capable of transporting bioactive molecules involved in cellular and interkingdom communication and can be engineered to improve targeting efficiency [84]. In addition, EVs display high bioavailability, resistance to degradation, and relatively low clearance rates, while their cargo can be modified to include therapeutic compounds, potentially improving drug delivery and reducing off-target toxicity [85]. One of the most influential studies in this field was published by Kamerkar et al. [86]. Using mouse models, including orthotopic xenografts and genetically engineered mouse models (GEMMs), the authors compared engineered liposomes with engineered exosomes derived from normal fibroblasts for the targeted treatment of pancreatic ductal adenocarcinoma (PDAC). These vesicles were loaded with siRNA or shRNA and designed to specifically target tumor cells. The results demonstrated that engineered exosomes showed superior cellular uptake compared with liposomes, largely due to the presence of CD47 on their surface, which also contributed to reduced clearance [86]. Several experimental studies have confirmed the potential of EV-based drug delivery in different tumor models. In glioblastoma cells, EVs conjugated with folic acid and loaded with temozolomide—the standard chemotherapeutic drug used for glioblastoma treatment—together with the flavonoid quercetin led to a stronger inhibition of cell proliferation and tumor angiogenesis compared with treatment using the drug alone [87]. Similar results have been observed in murine models of lung metastasis, where EVs loaded with paclitaxel (PTX) produced greater tumor inhibition than the free drug [88]. In another study, EVs carrying curcumin significantly reduced tumor growth in H1299 lung cancer models [89]. Additional evidence supporting the therapeutic potential of engineered EVs in lung cancer was provided by Amreddy et al. [90], who conducted an in vitro study on NSCLC cell lines (A549, HCC827, and H1299), as well as normal fibroblasts (MRC-9) and human embryonic kidney cells (HEK293). The researchers designed tumor-targeted multifunctional EVs (tt-Mfn-EVs) containing gold nanoparticles conjugated with cisplatin. To improve tumor targeting, the outer membrane of the vesicles was also modified with transferrin, a ligand for the transferrin receptor (TfR), which is overexpressed in lung cancer cells. The engineered vesicles produced a greater reduction in cell viability in A549 cells than in HCC827 cells, which express lower levels of TfR. Moreover, the treatment induced high levels of apoptosis and DNA damage in tumor cells while maintaining low toxicity in normal MRC-9 and HEK293 cells compared with cisplatin alone [90]. Another notable example is the study by Liu et al. [91], which explored the use of bovine milk-derived extracellular vesicles (mEVs) engineered for multimodal anticancer therapy. These vesicles were modified with two nanobodies—7D12 targeting EGFR and KN035 targeting Programmed Death-Ligand 1 (PD-L1)—and were loaded through electroporation with a miR-21-5p inhibitor. This design aimed to simultaneously target tumor cells and tumor-associated macrophages (TAMs). Experiments performed on several tumor cell lines derived from colorectal cancer (CRC), including HCT116, CT26, MKN45, SKMEL2, B16, and patient-derived tumor-like cell clusters (PTCs), showed that vesicles containing only the miR-21-5p inhibitor had lower uptake and weaker tumor inhibition than nanobody-modified mEVs carrying the same inhibitor. In addition, the latter induced a stronger repolarization of TAMs from the pro-tumoral M2 phenotype to the antitumoral M1 phenotype. The highest cellular uptake was observed in EGFR- and PD-L1-positive cells such as HCT116 and CT26, as well as in macrophage populations including THP-1, RAW264.7, and BMDM cells. In vivo experiments using BALB/c mice bearing CT26 colorectal tumors and C57BL/6 mice with B16 melanoma showed that engineered mEVs achieved approximately 85% tumor inhibition when used alone. When combined with radiotherapy or with an anti-PD-1 monoclonal antibody, tumor reduction reached approximately 94%. Both engineered and non-engineered mEVs showed low toxicity, and their combination with radiotherapy also induced significant immune remodeling of the tumor microenvironment [91]. Alternative EV sources have also been explored. Meliciano et al. [92] investigated EVs isolated from expired platelet concentrates, demonstrating how materials normally considered laboratory waste could be repurposed for biomedical applications. After loading these vesicles with paclitaxel, they were tested on breast cancer cells and human umbilical vein endothelial cells (HUVECs). The treatment resulted in a 70% reduction in tumor invasiveness and approximately a 30% decrease in both cell migration and angiogenesis, supporting the potential therapeutic value of platelet-derived EVs [92].

Although many studies have focused on EVs derived from human cells, these are not the only available source. Plants also release PDEVs which are increasingly being investigated as drug delivery systems. PDEVs can be used either after engineering or in their natural form. For example, Fang et al. [93] isolated vesicles from kiwifruit (KEVs) and loaded them with sorafenib, a drug commonly used to treat liver and kidney cancers. Experiments performed on Hepatoblastoma G2 (HepG2) cells showed higher cellular uptake, greater inhibition of proliferation, increased apoptosis, and lower toxicity compared with the free drug [93]. Similar findings were reported by Bhom et al. [94]. EVs isolated from *Aloe arborescens*, *Zingiber officinale*, and *Nigella sativa* were loaded with itraconazole and tested on glioblastoma cells (A172). The vesicles were able to cross the BBB more efficiently than the free drug, showed high cellular uptake, displayed preferential cytotoxicity toward tumor cells, and enabled prolonged drug release [94]. Overall, EVs offer several advantages for drug delivery, including high stability, biocompatibility, and low immunogenicity. Their membranes can be engineered to improve the delivery of therapeutic molecules into tumor cells. All the described information is summarized in Table 4.

## 5. Plant-Derived Extracellular Vesicle Effects on Cancer

**Figure 2:**
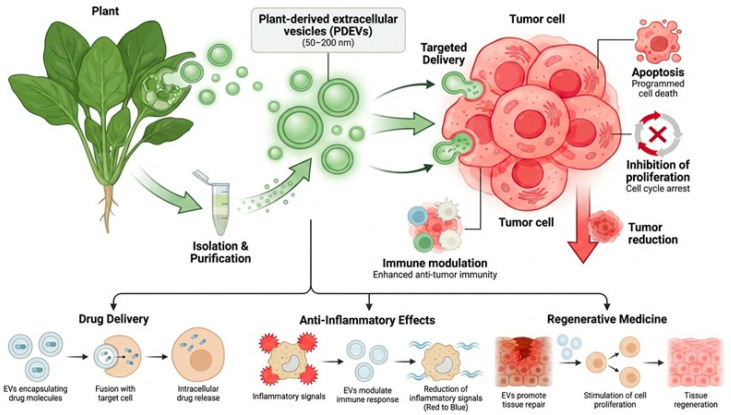
This image illustrates the therapeutic potential of PDEVs, emphasizing their applications in antitumor activity, drug delivery systems, anti-inflammatory effects, and regenerative medicine.

Plant extracts have therapeutic applications against various types of cancer [95]. In recent years, PDEVs have been shown to possess a range of therapeutic activities, including antitumor effects. In Figure 2, the properties of PDEVs are summarized.

Various studies have shown that PDEVs from different sources can induce cancer cell death, apoptosis, cell cycle arrest, and inhibit proliferation. For instance, Tajik et al. [96] observed that EVs derived from *Cannabis sativa*, which contain high levels of cannabidiol (CBD), reduced the viability of hepatocellular carcinoma cell lines, induced mitochondrial-dependent apoptosis, and inhibited the cell cycle in a dose- and time-dependent manner, while having no significant effects on the growth of normal cells (HUVECs). Another study conducted by Özkan et al. [97] highlights the potential of using garlic-derived EVs to treat kidney and lung cancer. Their findings provide evidence that these EVs can inhibit proliferation and induce apoptosis in lung (A549) and kidney (A498) cancer cells. Additionally, garlic-derived EVs have demonstrated apoptotic and antiproliferative activity against neuronal (SH-SY5Y), hepatoma (Hep3B), pancreatic adenocarcinoma (Panc-1), glioblastoma (U87), and prostate cancer cell lines [98]. Celery-derived EVs can downregulate PD-L1 expression in co-culture with lung cancer cells, thereby enhancing T cell immunity. Furthermore, when loaded with PTX, these EVs demonstrate improved tumor-targeting capacity in vivo and can increase the number of CD8+ T cells [99]. In the work of Wang et al. [100], ginger-derived EVs revealed high cytotoxicity against the murine melanoma cell line B16F10 in a concentration-dependent manner, whereas other types of PDEVs demonstrate high biocompatibility. Boccia et al. demonstrate that EVs derived from the hairy roots of Salvia dominica are effective against pancreatic (MIA PaCa-2) and breast (MCF-7) cancer cells, exhibiting greater activity than gemcitabine, while showing no adverse effects on non-tumor cells (HaCaT) [17]. Kim et al. found that plant sap-derived EVs, isolated from Dendropanax morbifera and *Pinus densiflora*, decrease the viability of BC cells (MDA-MB-231, MCF-7) and skin tumor cells (A431) [18]. Their findings indicate that low concentrations of D. morbifera EVs (5 µg/mL) selectively reduce the viability of cancer cells without affecting normal breast (MCF10A) and skin (HNF) cells. However, at higher concentrations (20–50 µg/mL), *P. densiflora* EVs increase the viability of normal breast cells, while both D. morbifera and *P. densiflora* EVs significantly decrease the viability of both cancerous and normal skin cells [18]. Furthermore, the study by Takakura et al. [19] suggests that nanovesicles derived from Citrus limon inhibit the growth of colorectal p53-inactivated cancer cells [19]. Anusha et al. [101]. demonstrated that ginger exosome-like nanoparticles (GELNs) reduce the growth of breast cancer cells (MDA-MB-231) in a dose-dependent manner, inhibit migration and colony formation, and induce apoptosis [101]. Similarly, perilla-derived EVs selectively inhibit BC cell proliferation and migration, especially in caveolin-1-high cells [102]. Chen et al. observed that tea flower EVs inhibit the migration, proliferation, and invasion of breast cancer cells [21]. These findings suggest that PDEVs, like plant extracts, have significant potential for cancer treatment. Nevertheless, further evaluation of different plant species is required, and additional studies are necessary—since most current research is preclinical—to determine optimal therapeutic concentrations in vitro and effective doses in vivo before these structures can be introduced into medical practice. In addition, future research should focus on developing and validating standardized, scalable protocols for PDEV isolation and characterization, ideally in accordance with MISEV recommendations. Comprehensive biodistribution and long-term safety studies in relevant animal models are also needed, along with integration of multi-omics profiling and advanced 3D tumor models to better link PDEV cargo with anticancer efficacy.

## 6. Extracellular Vesicles as Active Ingredients for Cancer Treatment

Tumor cells readily internalize small extracellular vesicles, which are secreted by all cell types and play an important role in intercellular communication. After their release into the extracellular environment, sEVs can be taken up by recipient tumor cells through different mechanisms, including membrane fusion, endocytosis, or micropinocytosis [99]. Vesicles produced by non-tumor sources may exert protective or anti-tumor effects. Plant-derived sEVs, for instance, have shown promising anti-cancer properties in several experimental models, while sEVs released by mesenchymal stem cells (MSCs) are known for their immunomodulatory functions and their ability to influence inflammatory responses and tumor progression [103,104,105]. This dual nature of EVs underscores the importance of carefully selecting their cellular source when considering their therapeutic use. Among the different types of EVs investigated for anti-cancer applications, those derived from natural killer (NK) cells have attracted considerable interest. NK cells are key components of the innate immune system and are well known for their ability to recognize and eliminate malignant cells. During their biogenesis, NK cell–derived sEVs incorporate a variety of cytotoxic molecules, including microRNAs, perforin, granzyme B, Fas ligand, and other tumor-killing factors that contribute to their anti-tumor activity. In a lung cancer mouse model, genetically engineered NK-derived sEVs demonstrated significant anti-tumor effects, reducing tumor growth while providing the advantages associated with cell-free therapies. Unlike adoptive cell therapies, EV-based approaches may reduce the risk of immune rejection or uncontrolled cell proliferation while still maintaining the cytotoxic potential of the parental immune cells [106]. Similar strategies have been explored in other cancer types. For example, NK-derived sEVs have been specifically engineered to enhance therapeutic efficacy in gastric cancer, where they were shown to improve anti-tumor activity and potentially increase the sensitivity of cancer cells to treatment [107]. These studies suggest that EVs derived from immune cells could represent a versatile platform for the development of innovative therapeutic strategies, either as standalone agents or as carriers for additional therapeutic molecules. Despite the encouraging results obtained with NK cell lines, vesicles derived from memory-like NK cells—particularly those originating from primary human NK cells—have been less extensively explored in cancer therapy. Nevertheless, recent studies indicate that these vesicles may possess unique functional properties. sEVs obtained from human memory-like NK cells cultured with interleukin (IL)-12, IL-15, and IL-18 have been shown to efficiently enter cancer cells through macropinocytosis. Once internalized, they can induce apoptosis via a caspase-dependent signaling pathway, ultimately leading to tumor cell death. These observations suggest that vesicles derived from memory-like NK cells could represent a novel class of therapeutic reagents with significant potential for cancer treatment [108]. Further molecular characterization of exosomes isolated from NK cells cultured with IL-2 or IL-15 revealed comparable vesicle production and a similar cargo composition. Detailed analyses of the molecules present inside these exosomes or exposed on their surface identified DNAX Accessory Molecule-1 (DNAM1), a receptor known to play a role in NK cell-mediated cytotoxicity. DNAM1 appears to be directly involved in the cytotoxic activity mediated by NK-derived exosomes, enabling them to exert their lytic effects specifically at tumor sites. This property supports the potential use of NK exosomes as tools in immunotherapy, where they could replicate some of the anti-tumor functions of NK cells while avoiding the challenges associated with cellular therapies [109]. In addition to inducing direct cytotoxicity, NK-derived EVs can also regulate gene expression in tumor cells. For example, exosomes released by NK cells have been reported to increase the expression of the microRNA let-7b-5p in pancreatic cancer cells. This upregulation results in the inhibition of tumor cell proliferation by targeting the cell cycle regulator CDK6, a key molecule involved in cell cycle progression. Through this mechanism, NK-derived vesicles can interfere with cancer cell growth and contribute to tumor suppression, providing further insight into how immune cell–derived EVs may counteract tumor development [110]. Moreover, it has been demonstrated that activated CD8+ T cell-derived EVs interrupt fibroblastic stroma-mediated tumor progression [111]. Dendritic cell-derived extracellular vesicles (DC-sEVs) have been explored for being used as a potential personalized vaccine in breast cancer. DC-sEVs showed significant advantages over dendritic cells, regarding efficacy, safety, storage, and potential delivery advantages, in vitro and in vivo. DC-sEVs have been shown to possess a higher abundance of Major Histocompatibility Complex molecules, which are crucial components of the adaptive immune system. Notably, DC-sEVs exhibit a capacity to suppress immune-cold breast cancer, a property not observed with the dendritic cells themselves [112]. Recently, EVs isolated from tumors are also finding application as vaccines. The study by Wang et al. [113] highlights the ability of EVs, particularly tumor-associated exosomes isolated from lung cancer cells, to enhance the activity of DC-based vaccines compared to vaccines consisting of DCs combined with traditional tumor lysates in vitro and in vivo. Indeed, these EVs deliver different antigens, demonstrating a greater capacity to strengthen the anti-cancer response mediated by CD8+ T cells and attenuate the immunosuppression of the tumor microenvironment [113]. Besides immune cell–derived vesicles, extracellular vesicles released by mesenchymal stem cells have also demonstrated significant therapeutic potential. MSC-derived EVs are widely studied because of their regenerative and immunomodulatory properties, which can influence multiple aspects of tumor biology. In a mouse model of breast cancer, EVs derived from human placenta MSCs significantly reduced tumor cell proliferation, migration, and colony-forming capacity. Interestingly, these inhibitory effects were observed without inducing apoptosis, suggesting that the vesicles primarily interfered with tumor cell growth dynamics and metastatic behavior rather than directly triggering cell death [114]. In addition, these EVs were found to exert a strong anti-angiogenic effect. In vitro experiments showed a marked reduction in angiogenic activity, accompanied by the downregulation of several angiogenesis-promoting genes in HUVEC. Consistent with these findings, in vivo analyses confirmed a significant inhibition of angiogenesis, indicating that MSC-derived EVs can modulate the tumor microenvironment by limiting the formation of new blood vessels necessary for tumor expansion and metastasis [114]. Overall, these investigations highlight the versatility of EVs as biologically active agents capable of modulating tumor progression through multiple mechanisms. Depending on their cellular origin and molecular composition, EVs can induce direct cytotoxic effects, regulate gene expression, modulate immune responses, and inhibit angiogenesis. These properties make them particularly attractive as active pharmaceutical ingredients for cancer therapy. Table 5 summarizes the described examples.

## 7. Conclusions

EVs have emerged as key mediators of intercellular communication and are increasingly recognized for their translational potential in oncology, particularly in cancer diagnosis and therapy. Over the past decade, extensive research has characterized EVs from diverse biological sources, including mammalian cells and plant-derived extracellular vesicles, highlighting their heterogeneity and source-dependent functional properties. Among current isolation strategies, differential ultracentrifugation remains the most widely applied approach and is still considered the reference method for EV purification, despite ongoing efforts to improve standardization and scalability. In cancer diagnostics, tumor-derived EVs are of particular interest because they carry molecular cargo—such as proteins, nucleic acids, and lipids—that reflects the biological state of their cells of origin. This has positioned EVs as promising biomarkers for liquid biopsy applications, offering a minimally invasive alternative to tissue-based sampling. Such approaches enable longitudinal monitoring of tumor dynamics, including disease progression and therapeutic response, with greater feasibility than conventional solid biopsies. From a therapeutic perspective, EVs are being actively explored as natural nanocarriers for drug delivery. Their intrinsic properties, including high biocompatibility, low immunogenicity, and structural stability conferred by a lipid bilayer, make them attractive compared with synthetic delivery systems. In addition, EVs can protect and transport a wide range of bioactive cargoes and can be further engineered to enhance targeting efficiency toward tumor tissues, thereby expanding their applicability in precision oncology. PDEVs have further broadened this field due to their availability from renewable sources and their reported biological activity in mammalian systems. Emerging evidence suggests that plant-derived vesicles may exert anti-cancer effects, although their molecular mechanisms are still being elucidated and remain incompletely understood. Preliminary findings, including the possibility that certain plant vesicles may contain complex intracellular components, remain speculative and require further validation before functional implications can be confirmed. Despite these promising advances, several critical barriers still limit clinical translation. The absence of standardized, reproducible, and scalable isolation and purification protocols continues to affect EV yield, purity, and comparability across studies [115]. Additional challenges include large-scale production, stability during storage, and robust quality control frameworks, all of which are essential for clinical-grade development. Nevertheless, accumulating preclinical evidence supports the therapeutic potential of EV-based approaches in oncology. Looking forward, underexplored biological sources such as marine organisms may provide additional diversity in EV populations and associated bioactive molecules. Given the rich pharmacological potential of marine-derived compounds, vesicles originating from these organisms could represent a novel avenue for identifying functional biomolecules with anti-tumor activity [116]. However, this area remains largely unexplored and warrants systematic investigation. In conclusion, EVs and PDEVs constitute a rapidly expanding research field with strong implications for cancer diagnosis and therapy. While significant methodological and translational challenges remain, continued progress in vesicle characterization, standardization, and functional engineering is expected to further clarify their role and accelerate their integration into future oncological applications.

## Figures and Tables

**Table 1:** Advantages and disadvantages of EV isolation methods.

Isolation Method	Advantages	Disadvantages	Reference
Differential ultracentrifugation (dUC)	Widely used and standardized; relatively inexpensive; provides consistent EV yields	Time-consuming; possible co-isolation of proteins with similar sedimentation properties; high centrifugal forces may damage vesicles or cause aggregation	[25,26,27]
Size-exclusion chromatography (SEC)	Simple and relatively inexpensive; allows separation based on particle size; reusable matrix; can be adapted for affinity purification	Slow process; difficult to scale up; requires specific equipment	[28]
Density gradient centrifugation (DGC)	High purity and homogeneous EV populations; effective when combined with dUC	Long processing time; difficult vesicle recovery from gradient; reduced yield; strong centrifugal forces may affect vesicle stability	[29,30]
Polymer precipitation (PEG-based)	Rapid; scalable; reproducible; relatively high yield	Co-precipitation of contaminants such as proteins; removal of polymer can be difficult and increase costs	[31,32,33,35]
Field-flow fractionation (FFF)	Gentle separation; good recovery; suitable for heterogeneous vesicle populations; produces relatively homogeneous fractions	Cannot distinguish between particle types; often requires combination with other techniques such as ultracentrifugation	[36]
Free-flow electrophoresis (FFE)	High-resolution separation based on isoelectric point; scalable method	Requires specialized equipment; still under investigation for routine EV isolation	[37,38]
Ion-exchange chromatography	Selective isolation of EV subpopulations based on membrane markers; useful for studying vesicle origin or function	Possible co-isolation of molecules with the same marker; may exclude EVs lacking the targeted marker; antibody-based approaches can be expensive	[39]
Enzymatic digestion + dUC (plant tissues)	High yield; reduced contamination from cytosolic RNA	Requires enzymatic treatment and additional preparation steps	[40]
Hydrophobic interaction chromatography (HIC)	Very low cost; rapid isolation; high impurity removal	Risk of bacterial contamination; difficulty distinguishing intra- and extracellular vesicles; limited scalability; lack of standardization	[41]
Tangential flow filtration (TFF)	Scalable; gentle process that preserves vesicle integrity; yields comparable to ultracentrifugation	Possible membrane fouling; vesicle loss due to adsorption to the membrane	[42]

**Table 2:** Overview of the main clinical trials involving extracellular vesicles. The search was conducted using the source https://clinicaltrials.gov/ (accessed on 16 April 2026) and writing cancer in the “Condition/disease” box, Biomarker in the “Other terms” box, and Extracellular vesicles in the “Intervention/treatment” box. Number of studies (*n*) = 28. N/A = Not available.

Trial ID	Study Type	Phase	Cancer Type	EV Source	EV Biomarker	Status
1. NCT07304856	Observational	N/A	Lymphangioleiomyomatosis (LAM)	Plasma	N/A	Ongoing
2. NCT03262311	Interventional	Not applicable	Carcinomas (head & neck, lung, bladder, cervix, breast)	Blood	N/A	Completed
3. NCT06104930	Interventional	Not applicable	Meningioma	Plasma	DNA	Ongoing
4. NCT06798805	Observational	N/A	Myeloproliferative Neoplasms	Blood and bone marrow	Protein	Active, not recruiting
5. NCT05831397	Observational	N/A	Breast cancer	Plasma	Protein	Ongoing
6. NCT07185360	Interventional	Not applicable	Hepatocellular carcinoma	Blood	N/A	Ongoing
7. NCT04523389	Observational	N/A	Colorectal cancer	Blood	miRNA	Terminated
8. NCT07491081	Interventional	Not applicable	Ovarian cancer	Blood	Protein	Ongoing
9. NCT05417048	Observational	N/A	Breast cancer	Serum	miRNA	Unknown
10. NCT07376512	Observational	N/A	NSCLC and melanoma	Blood	Proteins	Not yet recruiting
11. NCT05424029	Observational	N/A	NSCLC	Bronchial washing	N/A	Ongoing
12. NCT05798338	Observational	N/A	Breast cancer	Blood	Proteins	Ongoing
13. NCT04852653	Observational	N/A	Rectal cancer	Blood	DNA and protein	Ongoing
14. NCT06981377	Observational	N/A	Prostate cancer	Plasma	DNA, RNA and protein	Ongoing
15. NCT06278064	Observational	N/A	Gastrointestinal cancers	Plasma	Proteins	Unknown
16. NCT04298398	Observational	Not applicable	Head and neck cancer	Blood	N/A	Unknown
17. NCT07070011	Observational	N/A	Small Cell Lung Cancer (SCLC)	Blood	miRNA	Ongoing
18. NCT06764524	Observational	N/A	Hairy Cell Leukemia (HCL)	Plasma	N/A	Ongoing
19. NCT07355985	Observational	N/A	Prostate cancer	Urine	N/A	Completed
20. NCT04993378	Observational	N/A	Gastrointestinal cancers	Blood	N/A	Unknown
21. NCT07436416	Interventional	Not applicable	Kidney cancer	Blood and urine	miRNA	Completed
22. NCT05191849	Interventional	Not applicable	Small Cell Lung Cancer (SCLC)	Blood	Long RNA	Unknown
23. NCT06782854	Observational	N/A	Lymphomas	Plasma	N/A	Completed
24. NCT07500129	Observational	N/A	Breast cancer	Serum	miRNA	Not yet recruiting

**Table 3:** EVs as biomarkers.

Cancer Type	EV Biomarker Type	Key Characteristics	Reference
ccRCC	miR-210, miR-1233 (EpCAM+ EVs)	Elevated in patient serum EVs vs. controls; diagnostic sensitivity/specificity 70%/62% and 81%/76%	[43,44,45]
ccRCC	RNA panel (NDUFA4L2, SERPINA1, VEGFA, EGLN3, CPE, C6orf223; APOC1, TGFBI, LINC00887)	EV RNA signatures from Ti-EVs and uEVs; AUC 0.922 (low-grade), 0.874 (high-grade); decrease after tumor removal	[46]
Breast cancer	EDIL3 protein	EV protein associated with metastasis in circulating sEVs	[51]
Breast cancer	Fibronectin	EV surface marker proposed for ER+/ER− subtype discrimination	[52,53]
Breast cancer	CD47	Reduced CD47+ EVs in patients vs. healthy controls (0.7% vs. 10%)	[54]
Breast cancer	miRNAs (EV-associated)	Dysregulated miRNAs proposed as diagnostic biomarkers	[55]
Pancreatic cancer	KRAS mutant DNA in sEVs	Higher detection sensitivity than cfDNA (66.7% localized; 85% metastatic)	[56]
Pancreatic cancer	GPRC5C, EPS8 proteins	Enriched EV proteins in patient samples	[57]
Pancreatic cancer	lncRNA HULC	Promotes EMT, migration, and invasion via EV-mediated transfer	[58]
Lung cancer (NSCLC)	EGFR-mutant DNA in sEVs	Higher sensitivity/specificity than cfDNA, especially in the early stage	[59]
Lung cancer (APE/TPE/NPE)	miR-205-5p, miR-200 family, miR-203a-3p, miR-148a-3p, miR-451a, miR-150-5p	Differential miRNA expression distinguishes malignant vs. inflammatory vs. benign pleural disease	[60]
Lung cancer (NSCLC)	miRNA panel (miR-378a, miR-379, miR-139-5p, miR-200b-5p)	Screening signature; sensitivity 97.5%, specificity 72%	[61]
Lung cancer (NSCLC)	miRNA panel (miR-151a-5p, miR-30a-3p, miR-200b-5p, miR-629, miR-100, miR-154-3p)	Diagnostic signature; sensitivity 96%, specificity 60%	[61]
Lung cancer	miR-21, miR-155	Elevated in tumor cells, EVs, and recurrence models; associated with metastasis	[62]
Lung cancer (NSCLC)	Multi-protein EV panel (CD81, TAG72, TSG101, CD9, EGFR, MUC1, NY-ESO-1, CD151)	Combined diagnostic accuracy ~75.3%	[63]
Lung cancer	Salivary EV proteins (BPIFA1, CRNN, MUC5B, IQGAP)	Shared tumor signatures in saliva and lung EVs for non-invasive diagnosis	[64]
Pediatric high-grade glioma	GLAST+/PTPRZ1+ EV marker	Distinguishes tumor-derived EVs from astrocyte-derived EVs	[65]
H3K27M diffuse midline glioma	EV proteins, miRNAs, metabolites	Mediates radiation resistance via metabolic reprogramming and DNA repair enhancement	[66]
Glioblastoma	EGFRvIII mRNA (EV-derived)	Detectable in serum EVs; complements tissue biopsy; detects false negatives	[67]
Glioblastoma	miR-21, miR-15b-3p; EGFRvIII; circRNAs; CD44, GFAP	High diagnostic performance (87–100% sensitivity; 73–100% specificity)	[68]
Glioblastoma	EV enrichment strategies (SEC-CD44 IP, microfluidics, 5-ALA/PpIX)	Improves tumor-derived EV isolation and diagnostic specificity	[68]
Glioblastoma	EV-based comparison vs. CTCs/cfNAs	EVs outperform due to BBB crossing, stability, and higher molecular integrity	[69]
Prostate cancer	ERG, PCA3, SPDEF mRNA (EV-based test)	FDA-approved ExoDx Prostate IntelliScore for non-invasive diagnosis	[71]
Prostate cancer	Semen-derived EV transcripts	Predict tumor aggressiveness	[72]
CRPC	miRNAs, lncRNAs, proteins in EVs	Mediate ADT resistance and tumor progression via intercellular transfer	[73]
CRPC	EV-based diagnostic comparison	EVs outperform ctDNA/CTCs due to stability and abundance	[73]
Ovarian cancer	miR-1246, miR-1290, miR-483, miR-429, miR-34b-3p, miR-34c-5p, miR-145-5p, miR-449a	EV miRNAs promote migration/invasion; diagnostic potential	[75]
Ovarian cancer	RAB14, SCAMP3, FAM3C	EV proteins correlated with progression-free survival	[76]
Ovarian cancer	CORO1B, LAMP2, MSLN	Tumor-specific EV protein signature vs. healthy controls	[76]
Ovarian cancer	P15636 protease (bacterial-derived protein in EVs)	Potential early diagnostic biomarker in ascites EVs	[77]

**Table 4:** EVs as delivery systems.

Cancer Type/Model	EV Type Used as Drug Delivery System	Therapeutic Cargo/Engineering Strategy	Key Characteristics	Reference
Pancreatic ductal adenocarcinoma (PDAC)	Engineered exosomes derived from normal fibroblasts	siRNA or shRNA targeting tumor cells	Higher cellular uptake compared with engineered liposomes; CD47 on EV surface reduced immune clearance and improved delivery efficiency	[86]
Glioblastoma	Folic acid–conjugated EVs	Temozolomide + quercetin	Enhanced inhibition of tumor cell proliferation and angiogenesis compared with temozolomide alone	[87]
Lung metastasis (murine model)	Drug-loaded EVs	Paclitaxel (PTX)	Greater tumor inhibition compared with free paclitaxel	[88]
Lung cancer (H1299 model)	Drug-loaded EVs	Curcumin	Significant reduction in tumor growth compared with untreated controls	[89]
Non-small cell lung cancer (NSCLC)	Tumor-targeted multifunctional EVs (tt-Mfn-EVs)	Gold nanoparticles conjugated with cisplatin; membrane modified with transferrin (TfR targeting)	Increased apoptosis and DNA damage in tumor cells; greater cytotoxicity in TfR-expressing cells; low toxicity in normal cells	[90]
Colorectal cancer/melanoma models	Bovine milk-derived EVs (mEVs) engineered with nanobodies	miR-21-5p inhibitor; surface nanobodies targeting EGFR (7D12) and PD-L1 (KN035)	Enhanced cellular uptake; repolarization of tumor-associated macrophages (M2→M1); ~85% tumor inhibition alone and ~94% combined with radiotherapy or anti-PD-1 therapy	[92]
Breast cancer	Platelet-derived EVs from expired platelet concentrates	Paclitaxel	Reduced tumor invasiveness (~70%); decreased cell migration and angiogenesis (~30%)	[92]
Liver cancer model (HepG2 cells)	Plant-derived EVs from kiwifruit (KEVs)	Sorafenib	Higher cellular uptake, stronger proliferation inhibition, increased apoptosis, and lower toxicity than free drug	[93]
Glioblastoma	Plant-derived EVs from *Aloe arborescens*, *Zingiber officinale*, and *Nigella sativa*	Itraconazole	Efficient crossing of the BBB, preferential cytotoxicity toward tumor cells, high uptake, and prolonged drug release	[94]

**Table 5:** EVs as active ingredients.

Cancer Type/Model	EV Type Used as Active Ingredient	Source of Vesicles	Key Characteristics/Mechanism of Action	Reference
Lung cancer (mouse model)	Genetically engineered NK-derived sEVs	Natural killer (NK) cells	Reduced tumor growth through delivery of cytotoxic molecules such as perforin, granzyme B, Fas ligand, and miRNAs; cell-free anti-tumor therapy with lower risk of immune rejection.	[106]
Gastric cancer	Engineered NK-derived sEVs	Natural killer (NK) cells	Enhanced anti-tumor activity and increased sensitivity of cancer cells to treatment.	[107]
General cancer models	sEVs from memory-like NK cells	Primary human NK cells cultured with IL-12, IL-15, and IL-18	Efficient tumor cell entry via macropinocytosis and induction of caspase-dependent apoptosis.	[108]
General tumor models	NK-cell exosomes enriched in DNAM1	NK cells cultured with IL-2 or IL-15	DNAM1-mediated cytotoxicity enables targeted lytic activity at tumor sites, supporting immunotherapeutic applications.	[109]
Pancreatic cancer	NK-derived exosomes	Natural killer (NK) cells	Upregulation of let-7b-5p in tumor cells, leading to inhibition of proliferation through targeting of CDK6.	[110]
Fibroblastic stroma-mediated tumor progression model	CD8+ T cell-derived EVs	Activated CD8+ T cells	Interrupted fibroblastic stroma-mediated tumor progression and modulated the tumor microenvironment.	[111]
Breast cancer	Dendritic cell-derived sEVs (DC-sEVs)	Dendritic cells	Acted as a personalized vaccine platform; enriched in MHC molecules and capable of suppressing immune-cold breast cancer more effectively than dendritic cells alone.	[112]
Lung cancer vaccine model	Tumor-associated exosomes	Lung cancer cells	Enhanced dendritic cell vaccine efficacy, strengthened CD8+ T-cell anti-tumor responses, and reduced tumor microenvironment immunosuppression.	[113]
Breast cancer (mouse model)	MSC-derived EVs	Human placenta mesenchymal stem cells	Reduced tumor proliferation, migration, and colony formation; inhibited angiogenesis through downregulation of pro-angiogenic genes without inducing apoptosis.	[114]

## Data Availability

No new data were created or analyzed in this study. Data sharing is not applicable to this article.

## Abbreviations

The following abbreviations are used in this manuscript:

APE	lung adenocarcinoma
AUC	area under the curve
BBB	blood–brain barrier
BC	Breast Cancer
ccRCC	clear-cell Renal Cell Carcinoma
cfNAs	cell-free nucleic acids
CRPC	castration-resistant prostate cancer
CTCs	circulating tumor cells
DNAM1	DNAX Accessory Molecule-1
dUC	differential Ultracentrifugation
DGC	Density Gradient Centrifugation
EGFR	epidermal growth factor receptor
EVs	Extracellular vesicles
EXPOs	exocyst-positive organelles
FFE	Free-Flow Electrophoresis
FFF	Field-Flow Fractionation
GMP	Good Manufacturing Practice
HCC	hepatocellular carcinoma
HEK293	human embryonic kidney cells
HIC	Hydrophobic interaction chromatography
HULC	Highly Upregulated in Liver Cancer
HUVECs	human umbilical vein endothelial cells
ISEV	International Society for Extracellular Vesicles
IL	Interleukin
lEVs	large extracellular vesicles
mEVs	milk-derived extracellular vesicles
MISEV	Minimal Information for Studies of Extracellular Vesicles
MRC-9	normal fibroblasts
MSCs	mesenchymal stem cells
NK	natural killer
NPE	benign pleural lesions
NSCLC	non-small cell lung cancer
PBS	phosphate-buffered saline
PCa	Prostate Cancer
PDEVs	Plant-derived extracellular vesicles
PD-L-1	Programmed Death-Ligand 1
pDMG	pediatric diffuse midline gliomas
PEG	Polyethylene glycol
pHGG	pediatric High-Grade Glioma
PTX	Paclitaxel
SCLC	small cell lung cancer
sEVs	small extracellular vesicles
SEC	Size Exclusion Chromatography
TFF	Tangential flow filtration
TfR	transferrin receptor
Ti-EVs	tumor Tissue Extracellular vesicles
TPE	pleural tuberculosis
uEVs	urine Extracellular vesicles
